# Barthel Index is a valid and reliable tool to measure the functional independence of cancer patients in palliative care

**DOI:** 10.1186/s12904-022-01017-z

**Published:** 2022-07-12

**Authors:** Vinício dos Santos Barros, Daniela Bassi-Dibai, César Leonardo Ribeiro Guedes, Daniel Nunes Morais, Sabrina Marinho Coutinho, Gabriel de Oliveira Simões, Letícia Padilha Mendes, Plínio da Cunha Leal, Almir Vieira Dibai-Filho

**Affiliations:** 1 Postgraduate Program in Physical Education, Universidade Federal do Maranhão, São Luís, MA Brazil; 2 Pain and Palliative Care Sector, Maranhão Cancer Hospital, São Luís, MA Brazil; 3 Postgraduate Program in Programs Management and Health Services, Universidade Ceuma, São Luís, MA Brazil; 4 Department of Physical Education, Universidade Federal do Maranhão, São Luís, MA Brazil; 5 Postgraduate Program in Adult Health, Universidade Federal do Maranhão, São Luís, MA Brazil

**Keywords:** Functional capacity, Surveys and questionnaires, Cancer

## Abstract

**Background:**

Our objective was to verify the reliability, internal consistency and construct validity of the Barthel Index in Brazilian cancer patients in palliative care.

**Methods:**

We included patients with cancer, both sexes, and age greater than or equal to 18 years. We used to evaluate patients the Barthel Index, Karnofsky Performance Scale (KPS), and European Organization for Research in the Treatment of Cancer Questionnaire-core 15 (EORTC-QLQ-C15-PAL). The measurement properties evaluated in this study were test–retest and inter-rater reliability and construct validity (tested by means of correlations with other instruments).

**Results:**

We included 220 patients for construct validity and a subsample of 27 patients for reliability analyses. We observed adequate reliability (intraclass correlation coefficient ≥ 0.962) and internal consistency (Cronbach’s alpha = 0.942). There were adequate correlations between the Barthel Index and the KPS (rho = 0.766), and the functional capacity domain of the EORTC-QLQ-C15-PAL (rho = -0.698).

**Conclusion:**

The Brazilian version of the Barthel Index presents adequate test–retest and inter-rater reliability, acceptable internal consistency, and valid construct for measuring functional independence in cancer patients.

**Supplementary Information:**

The online version contains supplementary material available at 10.1186/s12904-022-01017-z.

## Background

Quality of life and quality of care are health aspects commonly evaluated in cancer patients in palliative care [1, 2]. In addition to these measures, the assessment of the functional status is common in the clinical routine of these patients as it indicates the patient’s ability to maintain independence through their performance in activities of daily living [3, 4]. In complement, the functional status has potential applications in survival predictions [3].

The Karnofsky Performance Scale (KPS) is one of the most used tools in cancer patients in palliative care to measure functional status. This scale was proposed in a book in 1949, consisting of 11 items ranging from 0 (full well-being) to 100% (death) [5]. The score can be categorized as follows: Group A (100%–80%), patient can perform daily activities independently; Group B (70%–50%), patient can perform daily activities with help; Group C (< 40%), patient requires continuous assistance and approaches death progressively [1, 6].

Another tool commonly used in the rehabilitation field is the Barthel Index. This tool was created in 1965 to assess functional independence to perform 10 daily activities [7]. Cross-cultural adaptations and validations of the Barthel Index are mostly performed on patients with neurological diseases and the elderly [8–12]. To the best of our knowledge, there is no study verifying the measurement properties of the Barthel Index in cancer patients. Of the studies carried out in this population, most of the literature presents observational studies measuring functional independence, such as in patients with cancer in hospice [13], with brain tumour [14], and with malignant spinal cord compression [15].

In this sense, considering the relevance of the Barthel Index, the present study aimed to verify the reliability, internal consistency and construct validity of this instrument in Brazilian cancer patients in palliative care.

## Methods

### Study design and ethical aspects

This is a cross-cultural study carried out according to the COnsensus-based Standards for the selection of health Measurement INstruments (COSMIN) [16]. The research was conducted in the Pain and Palliative Care sector of the Hospital do Câncer do Maranhão (São Luís, Northeast Brazil).

The study procedures were approved by the Research Ethics Committee of the Universidade Federal do Maranhão (opinion number 2.984.884) and followed all universal ethical principles. All participants signed a free and informed consent form and were 18 years old or older.

### Participants

We used the COSMIN recommendation of 100 patients as the minimum sample size for validity analyses [17]. A subsample of 27 patients was evaluated by two examiners at two moments (with an interval of 1 week) for reliability analyses. The eligibility criteria were: ≥ 18 years old; both sexes; cancer diagnosis confirmed by biopsy; no diagnosed cognitive changes; awareness of the cancer diagnosis; and ability to read and understand Brazilian Portuguese.

### Barthel Index

Functional independence was assessed using the Barthel Index, validated for Brazilian Portuguese in cerebrovascular diseases [12] and elderly [11]. The index analyzes 10 aspects: bowels, bladder, grooming, toilet use, feeding, transfer, mobility, dressing, stairs, and bathing. The total score ranges from 0 to 100 points. The higher the score, the greater the functional independence.

### Other clinical tools

To determine construct validity by means of correlations, in addition to the Barthel Index, we use the following instruments: The European Organization for Research in the Treatment of Cancer Questionnaire-core 15 (EORTC-QLQ-C15-PAL) and the KPS.

EORTC-QLQ-C15-PAL was validated for Brazilian Portuguese [18] and consists of 15 items distributed in three domains: functional scale (five items), symptom scale (nine items), and global health status (one item). For interpretation, each domain must be analyzed separately. The total score ranging from 0 to 100. The higher the score, the higher the overall health status, and the worse the symptoms and the functional capacity.

Functional status was assessed using the KPS [19]. The scale is scored from 0 to 100%. The higher the score, the higher functional capacity. This scale checks from signs of illness, difficulty to perform activities, need for help to perform activities, to total disability and death.

### Statistical analysis

Sociodemographic and clinical data are presented as the mean and standard deviation (SD) or as the absolute number and percent.

Internal consistency was calculated using Cronbach’s alpha and we considered values ≥ 0.70 as an indication of good internal consistency [17]. Test–retest and inter-rater reliability was assessed with 2 examiners and 2 assessments (interval of 7 days between assessments). The intraclass correlation coefficient (ICC), 95% confidence interval (CI), standard error of measurement (SEM), minimum detectable difference (MDD), and coefficient of variation (CV) were used to assess the reliability of the Barthel Index [20]. For the interpretation of the ICC value, we considered values ≥ 0.75 as suitable [21]. The agreement between measurements was analyzed using Bland–Altman plots.

For the correlations between the questionnaires, the normality of the data was initially verified using the Kolmogorov–Smirnov test. To determine construct validity, Spearman’s correlation coefficient (rho) was used to determine the magnitude of the correlation between the Barthel Index, the EORTC-QLQ-C15-PAL, and the KPS. We assume the following hypotheses: correlation greater than 0.50 (similar construct) between the Barthel Index, the functional capacity domain of the EORTC-QLQ-C15-PAL and the KPS; and correlation between 0.30–0.50 (related but different constructs) between the Barthel Index and the symptom and global health status domains of the EORTC-QLQ-C15-PAL [16].

All analysis was performed using SPSS version 17.0 (SPSS Inc., Chicago, IL, USA), with a 5% significance level was adopted.

## Results

Data collection started in October 2018 and ended in January 2022. Two hundred and twenty cancer patients were included for the construct validity analysis. The average age of these patients was 55.54 years (SD = 15.12) and the average treatment time was 9.85 months (SD = 9.52). A total of 27 patients completed the reliability phase. Table 1 shows the other personal and clinical characteristics of the study participants. Regarding to the other clinical variables, Table 2 presents the scores for the Barthel Index, the KPS, and the EORTC-QLQ-C15-PAL.

**Table 1:** Descriptive analysis of patients’ personal and clinical characteristics (*n* = 220)

Variables	Number (%)
Sex	
Male	111 (50.5)
Female	109 (49.5)
Marital status	
Single	50 (22.7)
Married	116 (52.7)
Widower	33 (15.0)
Divorced	21 (9.5)
Educational level	
Elementary	79 (35.9)
Basic	69 (31.4)
High school	65 (29.5)
Higher education	7 (3.2)
Professional activity	
Active	143 (65)
Inactive	77 (35)
Cancer: primary site	
Uterus	39 (17.7)
Stomach	32 (14.5)
Lung	25 (11.4)
Prostate	20 (9.1)
Liver	19 (8.6)
Leukemia	15 (6.8)
Mama	10 (4.5)
Kidney	9 (4.1)
Brain	7 (3.2)
Pancreas	6 (2.7)
Lymphoma	6 (2.7)
Esophagus	5 (2.3)
Osteosarcoma	5 (2.3)
Penis	4 (1.8)
Multiple myeloma	4 (1.8)
Others	14 (6.4)
Type of treatment	
Palliative	110 (50)
Curative	72 (32.7)
Both	38 (17.3)
Current treatment	
Drug therapy	117 (53.2)
Chemotherapy	69 (31.4)
Radiotherapy	11 (5.0)
Surgical	23 (10.5)
Metastasis	
Yes	124 (56.4)
No	96 (43.6)

**Table 2:** Descriptive analysis of clinical evaluations (*n* = 220)

Variables	Mean (standard deviation)
Barthel Index (score)	72.62 (19.95)
KPS (score)	61.68 (16.45)
EORTC-QLQ-C15-PAL (score)	
Functional	44.27 (25.26)
Symptoms	36.88 (20.35)
Quality of life	55.53 (28.90)

*KPS* Karnofsky Performance Scale, *EORTC-QLQ-C15-PAL*, European Organization for Research in the Treatment of Cancer Questionnaire-core 15

Regarding the construct validity, as shown in Table 3, we confirmed our hypothesis by observing a correlation magnitude greater than 0.50 between the Barthel Index, the KPS (rho = 0.766), and functional capacity domain of the EORTC-QLQ-C15-PAL (rho = -0.698). Furthermore, the correlations with the other domains of the EORTC-QLQ-C15-PAL were also satisfactory.

**Table 3:** Correlation between the score of the Barthel Index and the other study variables (*n* = 220)

Variables	Barthel Index
KPS	rho = 0.766, *p* < 0.001
EORTC-QLQ-C15-PAL	
Functional	rho = -0.698, *p* < 0.001
Symptoms	rho = -0.540, *p* < 0.001
Quality of life	rho = 0.748, *p* < 0.001

*KPS* Karnofsky Performance Scale, *EORTC-QLQ-C15-PAL* European Organization for Research in the Treatment of Cancer Questionnaire-core 15

**Fig. 1:**
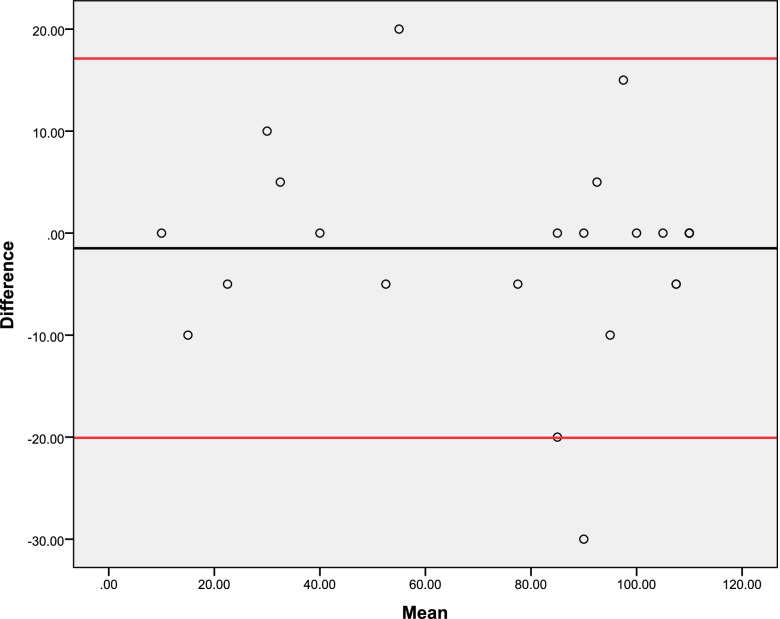
Bland–Altman plot of agreement between test–retest measures (*n* = 27)

**Fig. 2:**
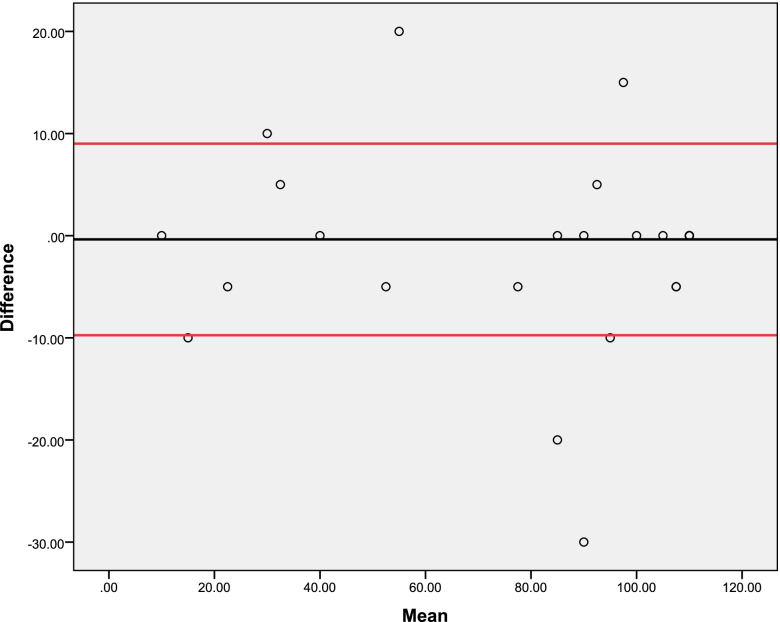
Bland–Altman plot of agreement between inter-rater measures (*n* = 27)

There was adequate test–retest and inter-rater reliability of the Barthel Index, with ICC ≥ 0.962, SEM ≤ 8.35%, and CV ≤ 3.99%, as shown in Table 4. There was also adequate internal consistency (Cronbach’s alpha = 0.942). Bland–Altman plots show adequate agreement between test–retest (Fig. 1) and inter-rater (Fig. 2) measures.

**Table 4:** Reliability and internal consistency of the Barthel index (*n* = 27)

Reliability and internal consistency	Values
Examiner 1	
Mean (standard deviation) of the test	79.25 (33.90)
Mean (standard deviation) of the retest	80.74 (34.63)
Examiner 2	
Mean (standard deviation) of the test	79.62 (34.30)
Mean (standard deviation) of the retest	81.11 (34.92)
Test–retest reliability	
ICC	0.962
95% CI	0.918 to 0.982
SEM (score)	6.68
SEM (%)	8.35
MDD (score)	18.51
MDD (%)	23.14
CV (%)	3.99
Inter-rater reliability	
ICC	0.990
95% CI	0.978 to 0.995
SEM (score)	3.41
SEM (%)	4.29
MDD (score)	9.45
MDD (%)	11.90
CV (%)	2.49
Internal consistency	
Cronbach’s alpha	0.942

*ICC* Intraclass correlation coefficient, *CI* Confidence interval, *SEM* Standard error of measurement, *MDD* Minimal detectable difference, *CV* Coefficient of variation

## Discussion

Our results showed that the Barthel Index is a reliable measure, with adequate internal consistency and valid to measure the functional independence of cancer patients in palliative care. The cross-cultural adaptation of the Barthel Index for elderly Brazilians conducted by Minosso et al. [11] showed similar results: adequate internal consistency (Cronbach's alpha = 0.90), but slightly lower than the value found in the present study (Cronbach's alpha = 0.942); valid construct based on the correlation with the Functional Independence Measurement (rho = 0.64), but slightly lower than the values found in the present study (rho ≥ 0.698). The ICC value was not calculated in this study [11], but our study found adequate values (ICC ≥ 0.962).

In addition to the Barthel Index, our study used the KPS and the EORTC-QLQ-C15-PAL as they are specific instruments for patients in palliative care and encompass the functional status domain. We did not use the Functional Independence Measurement to validate the construct, as did the study conducted by Minosso et al. [11], due to the absence of studies validating Functional Independence Measurement in cancer patients in palliative care. In addition, the Brazilian version of this instrument was validated in patients with spinal cord or brain injury [22].

The Barthel index has a broad context, so our study focused on cancer patients in palliative care. According to the COSMIN [16], the measurement properties of questionnaires or scales must be evaluated in different populations, as these tools may assume different psychometric behaviors. In this sense, other studies have validated the Barthel Index for other populations. A study carried out with the Brazilian population investigated the measurement properties of the Barthel Index in intensive care unit discharge and observed adequate internal consistency (Cronbach's alpha = 0.81), acceptable reliability (ICC = 0.98), and moderate to high correlations with other physical functioning measurement instruments (rho = 0.57 to 0.88) [23].

Study carried out with patients with Parkinson’s disease observed adequate internal consistency (Cronbach's alpha = 0.81), acceptable reliability (ICC ≥ 0.993) and moderate correlations of the Barthel Index score with the mobility (rho = -0.661) and activities of daily living (rho = -0.589) domains of the Parkinson’s Disease Questionnaire [24]. Investigation carried out with patients with respiratory diseases identified high reliability (ICC ≥ 0.93), good internal consistency (Cronbach's alpha = 0.89), and strong concurrent validity of the Barthel Index score with 6-min walking distance (r = -0.538) and Medical Research Council (rho = 0.70) [25].

In cancer patients, a recent cohort study points to the importance of the Barthel Index as a tool for predicting survival time in young and middle-aged adults with newly diagnosed gastric, colorectal and lung cancer [3]. In addition, a study conducted by Brazil et al. [14] highlight the high correlation between the Barthel Index and the KPS (rho = 0.872). However, although psychometrically adequate and strongly correlated, the Barthel Index and the KPS have their own characteristics.

Compared to the KPS, the Barthel Index has the following advantages: instrument specifically focused on daily activities (such as feeding, bathing, grooming, and toilet use), and presents important items for complete well-being that are not covered by the KPS, such as bowel and bladder control, toilet use, transfers (bed to chair and back), walking, and ascending and descending stairs. However, the KPS has greater scientific support for its use in palliative care patients, in addition to presenting adequate correlations with the prognosis of survival [26].

The Palliative Performance Scale (PPS) is another instrument with characteristics partially similar to the Barthel Index. This scale was developed in 1996 and was adapted from the KPS as a novel tool to quantify the performance status of patients receiving palliative care. The PPS items investigate the ambulation, activity and evidence of disease, self-care, intake, and conscious level. The PPS score correlates strongly with the KPS score, especially in patients with good clinical performance [19]. Similar to the comparisons made with the KPS, the Barthel Index presents as positive points compared to the PPS a more detailed approach to some functional aspects, especially bowel and bladder control, toilet use, transfers (bed to chair and back), walking, and ascending and descending stairs.

The present study has limitations that must be considered. Responsiveness was not evaluated as it required a longitudinal design study. Our entire sample consisted of patients in hospital care; thus, the measurement properties of patients in home or outpatient palliative care need to be investigated by future studies.

## Conclusion

The Brazilian version of the Barthel Index presents adequate test–retest and inter-rater reliability, acceptable internal consistency, and valid construct for measuring functional independence in cancer patients in palliative care.

## Supplementary Information

**Additional file 1.** 

**Additional file 2.** 

## Data Availability

Data available in supplementary file.
